# Erratum

**DOI:** 10.4269/ajtmh.19-0286err

**Published:** 2019-09

In an article published in the August 2019 issue on pages 319–322, “Case Report: Typhoid Fever Complicated by Acute Respiratory Distress Syndrome in a Pediatric Traveler” by Birabaharan and others (http://www.ajtmh.org/content/journals/10.4269/ajtmh.19-0286) the print and online versions are not identical. After further review of the case reports reviewed in the literature, the authors made adjustments to the results that they believe more accurately reflects previous cases of Typhoid Fever complicated by ARDS. These changes were made to the online version of the article but were not incorporated into the print version due to time constraints. The online version is correct.

This is an open-access article distributed under the terms of the Creative Commons Attribution License, which permits unrestricted use, distribution, and reproduction in any medium, provided the original author and source are credited.

